# (*E*)-1-(2-Amino­phen­yl)-3-(3,4,5-trimeth­oxy­phen­yl)prop-2-en-1-one

**DOI:** 10.1107/S1600536811033861

**Published:** 2011-08-27

**Authors:** Suchada Chantrapromma, Pumsak Ruanwas, Hoong-Kun Fun

**Affiliations:** a Crystal Materials Research Unit, Department of Chemistry, Faculty of Science, Prince of Songkla University, Hat-Yai, Songkhla 90112, Thailand; b Department of Chemistry and Center of Excellence for Innovation in Chemistry, Faculty of Science, Prince of Songkla University, Hat-Yai, Songkhla 90112, Thailand; c X-ray Crystallography Unit, School of Physics, Universiti Sains Malaysia, 11800 USM, Penang, Malaysia

## Abstract

In the asymmetric unit of the title chalcone derivative, C_18_H_19_NO_4_, there are three crystallographically independent mol­ecules (mol­ecules *A*, *B* and *C*). In mol­ecule *A*, the dihedral angle between two benzene rings is 12.22 (10)° and the plane of the central prop-2-en-1-one unit makes dihedral angles of 11.02 (13) and 2.64 (12)° with the two adjacent benzene rings. The corresponding angles in mol­ecule *B* are 12.35 (10), 18.78 (12) and 7.29 (12)°, respectively, and those in mol­ecule *C* are 15.40 (10), 15.62 (3) and 3.19 (13)°. In each mol­ecule, an intra­molecular N—H⋯O hydrogen bond generates an *S*(6) ring motif. In the crystal structure, the mol­ecules *B* are linked by inter­molecular N—H⋯O hydrogen bonds into a zigzag chain along the *c* axis, while the mol­ecules *A* and *C* are linked together *via* an N—H⋯O hydrogen bond into a dimer. Adjacent dimers are further connected by N—H⋯N hydrogen bonds into a three-dimensional network. Weak C—H⋯O and C—H⋯π inter­actions are also observed.

## Related literature

For bond-length data, see: Allen *et al.* (1987 ▶). For details of hydrogen-bond motifs, see: Bernstein *et al.* (1995 ▶). For related structures, see: Fun *et al.* (2010 ▶); Suwunwong, Chantrapromma & Fun (2009 ▶); Suwunwong, Chantrapromma, Pakdeevanich & Fun (2009 ▶). For background to and applications of chalcones, see: Batt *et al.* (1993 ▶); Gacche *et al.* (2008 ▶); Isomoto *et al.* (2005 ▶); Khatib *et al.* (2005 ▶); Nowakowska *et al.* (2001 ▶); Rojas *et al.* (2002 ▶); Shibata (1994 ▶); Sivakumar *et al.* (2007 ▶); Tewtrakul *et al.* (2003 ▶); Tomar *et al.* (2007 ▶). For the stability of the temperature controller used in the data collection, see: Cosier & Glazer (1986 ▶).
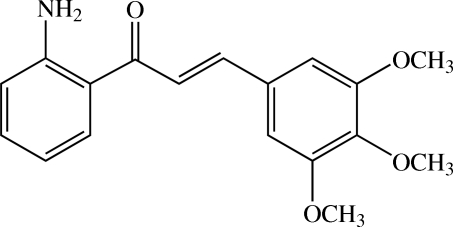


## Experimental

### 

#### Crystal data

C_18_H_19_NO_4_
*M* *_r_* = 313.34
Monoclinic, 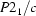

*a* = 14.8537 (3) Å
*b* = 20.5009 (4) Å
*c* = 19.5952 (3) Å
β = 127.043 (1)°
*V* = 4762.78 (16) Å^3^
*Z* = 12
Mo *K*α radiation
μ = 0.09 mm^−1^
*T* = 100 K
0.40 × 0.20 × 0.14 mm

#### Data collection

Bruker APEXII CCD area-detector diffractometer
Absorption correction: multi-scan (*SADABS*; Bruker, 2005 ▶) *T*_min_ = 0.964, *T*_max_ = 0.987
48383 measured reflections
10835 independent reflections
6967 reflections with *I* > 2σ(*I*)
*R*_int_ = 0.048

#### Refinement

*R*[*F* ^2^ > 2σ(*F* ^2^)] = 0.065
*wR*(*F* ^2^) = 0.124
*S* = 1.02
10835 reflections
631 parameters
H-atom parameters constrained
Δρ_max_ = 0.29 e Å^−3^
Δρ_min_ = −0.24 e Å^−3^

### 

Data collection: *APEX2* (Bruker, 2005 ▶); cell refinement: *SAINT* (Bruker, 2005 ▶); data reduction: *SAINT*; program(s) used to solve structure: *SHELXTL* (Sheldrick, 2008 ▶); program(s) used to refine structure: *SHELXTL*; molecular graphics: *SHELXTL*; software used to prepare material for publication: *SHELXTL* and *PLATON* (Spek, 2009 ▶).

## Supplementary Material

Crystal structure: contains datablock(s) global, I. DOI: 10.1107/S1600536811033861/is2761sup1.cif

Structure factors: contains datablock(s) I. DOI: 10.1107/S1600536811033861/is2761Isup2.hkl

Supplementary material file. DOI: 10.1107/S1600536811033861/is2761Isup3.cml

Additional supplementary materials:  crystallographic information; 3D view; checkCIF report

## Figures and Tables

**Table 1:** Hydrogen-bond geometry (Å, °) *Cg*1, *Cg*2 and *Cg*4 are the centroids of the C1*A*–C6*A*, C10*A*–C15*A* and C10–C15*B* rings, respectively.

*D*—H⋯*A*	*D*—H	H⋯*A*	*D*⋯*A*	*D*—H⋯*A*
N1*A*—H19⋯O1*A*	0.92	1.91	2.618 (3)	133
N1*A*—H20⋯N1*C*^i^	0.89	2.41	3.262 (3)	162
N1*B*—H21⋯O1*B*	0.88	1.96	2.634 (3)	132
N1*B*—H22⋯O4*B*^ii^	0.87	2.22	3.022 (3)	153
N1*C*—H23⋯O1*C*	0.93	1.93	2.633 (3)	130
N1*C*—H24⋯O3*A*^iii^	0.84	2.19	2.977 (2)	156
C15*B*—H15*B*⋯O1*A*	0.95	2.55	3.434 (3)	154
C18*B*—H18*D*⋯O3*C*^iv^	0.98	2.38	3.212 (3)	142
C18*B*—H18*F*⋯O1*A*	0.98	2.53	3.177 (3)	123
C2*B*—H8⋯*Cg*1	0.95	2.75	3.342 (2)	121
C2*C*—H14⋯*Cg*2^v^	0.95	2.94	3.674 (2)	135
C16*C*—H16*I*⋯*Cg*4^vi^	0.98	2.80	3.724 (2)	157

Symmetry codes: (i) 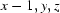; (ii) 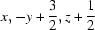; (iii) 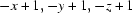; (iv) 
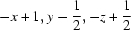
; (v) 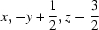; (vi) 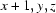.

**Fig. 1.**
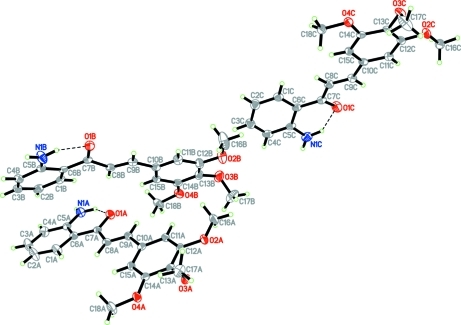
The molecular structure of the title compound, showing 50% probability displacement ellipsoids and the atom-numbering scheme. N—H···O hydrogen bonds are shown as dash lines.

**Fig. 2.**
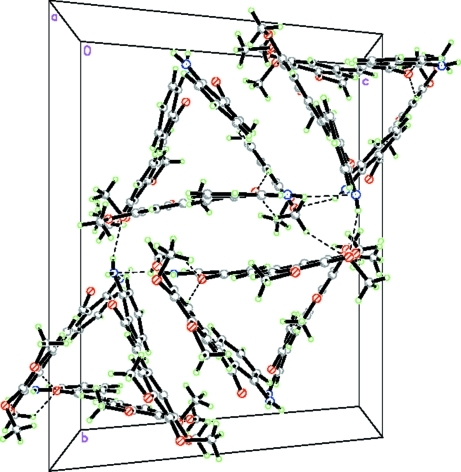
The crystal packing of the title compound viewed approxinately along the b-axis showing a 3D network. Hydrogen bonds are shown as dashed lines.

## supplementary crystallographic information

### Comment

Chalcones represent an important group of natural and synthetic compounds and posses a wide variety of pharmacological activities. Earlier research also proved that chalcones have antitubercular (Sivakumar *et al.*, 2007), antioxidant (Gacche *et al.*, 2008), antibacterial (Nowakowska *et al.*, 2001; Isomoto *et al.*, 2005), antifungal (Tomar *et al.*, 2007) and anticancer activities (Shibata, 1994) as well as HIV-1 protease inhibitory (Tewtrakul *et al.*, 2003), tyrosinase inhibitory (Khatib *et al.*, 2005) and nitric oxide inhibitory (Rojas *et al.*, 2002) and interleukin-1 (Batt *et al.*, 1993) properties. As our ongoing research on antibacterial activities and tyrosinase inhibitory proeprties of aryl/heteroaryl chalcones, we have previously reported the crystal structures of (*E*)-1-(4-bromophenyl)-3-(3,4,5-trimethoxyphenyl)prop-2-en-1-one (Suwunwong, Chantrapromma & Fun, 2009), (*E*)-1-(2-thienyl)-3-(3,4,5-trimethoxyphenyl)prop-2-en-1-one (Suwunwong, Chantrapromma, Pakdeevanich & Fun, 2009) and (*E*)-1-(2-furyl)-3-(3,4,5-trimethoxyphenyl)prop-2-en-1-one (Fun *et al.*, 2010). In the course of this work, we have synthesized the title compound (I) in order to compare their antibacterial activities and tysosinase inhibitory properties. However our experiment shows that (I) doesn't exhibit both antibacterial and tyrosinase inhibitory activities. Herein the crystal structure is reported.

There are three crystallographically independent molecules *A*, *B* and *C* in the asymmetric unit of (I) with differences in bond angles (Fig. 1). The molecular structure of (I), C_18_H_19_NO_4_ is twisted with the the dihedral angle between the C1–C6 and C10–C15 benzene rings being 12.22 (10)° in molecule *A* whereas it is 12.35 (10) and 15.40 (10)° in molecules *B* and *C*, respectively. The central prop-2-en-1-one bridge (C7–C9/O1) in molecule *A* is planar whereas in molecules *B* and *C* it is slightly twisted which can be indicated by the torsion angle O1–C7–C8–C9 = 1.4 (3), 7.6 (3) and -3.4 (3)° in molecules *A*, *B* and *C*, respectively. The mean plane through this bridge makes the dihedral angles of 11.02 (13) and 2.64 (12)° with the two adjacent C1–C6 and C10–C15 benzene rings, respectively, in molecule *A*, whereas the corresponding values are 18.78 (12) and 7.29 (12)° in molecule *B*, and 15.62 (3) and 3.19 (13)° in molecule *C*. The three methoxy groups of the 3,4,5-trimethoxyphenyl unit have two different orientations: the two methoxy groups at the *meta*-positions (at atom C12 and C14 positions) are co-planar with the attached benzene ring with torsion angles C16–O2–C12–C11 = -0.2 (3)° and C18–O4–C14–C13 = -177.75 (19)° whereas the third one at *para*-position (at atom C13) is out of plane with the torsion angle C17–O3–C13–C12 = 102.8 (2)°; the corresponding values are -8.1 (3), 173.43 (18) and 92.6 (3)° in molecule *B*; and -0.9 (3), -172.62 (16) and -106.7 (2)° in molecule *C*. In each molecule, an intramolecular N—H···O hydrogen bond (Table 1) generates an S(6) ring motif (Bernstein *et al.*, 1995). The bond distances agree with the literature values (Allen *et al.*, 1987) and are comparable with the related structures (Fun *et al.*, 2010; Suwunwong, Chantrapromma & Fun, 2009; Suwunwong, Chantrapromma, Pakdeevanich & Fun, 2009).

In the crystal packing (Fig. 2), the molecules B are linked by intermolecular N—H···O hydrogen bonds (Table 1) into a zigzag chain along the *c* axis, while the molecules A and C are linked together via an N—H···O hydrogen bond into a dimer. Adjacent dimers are further connected by N—H···N hydrogen bonds (Table 1) into a three-dimensional network. The crystal is stabilized by intermolecular N—H···O and N—H···N hydrogen bonds together weak C—H···O and C—H···π interactions (Table 1).

### Experimental

The title compound was synthesized by dissolving the 3,4,5-trimethoxybenzaldehyde 0.5 g (2.55 mmol) in ethanol (20 ml). 2-aminoacetophenone 0.31 ml (2.55 mmol) and 30% NaOH aqueous solution (5 ml) were then added. The mixture was stirred at room temperature for 2 hr. A yellow precipitate was formed and was then filtered, washed with distilled water and dried in vacuum. Yellow block-shaped single crystals of the title compound suitable for *x*-ray structure determination were recrystalized from ethanol by the slow evaporation of the solvent at room temperature after a week, Mp. 391–393 K.

### Refinement

All H atoms were positioned geometrically and allowed to ride on their parent atoms, with *d*(N—H) = 0.84–0.93 Å, *d*(C—H) = 0.95 Å for aromatic and CH, and *d*(C—H) = 0.98 Å for CH_3_. The *U*_iso_ values were constrained to be 1.5*U*_eq_ of the carrier atom for methyl H atoms and 1.2*U*_eq_ for the remaining H atoms. A rotating group model was used for the methyl groups. The highest residual electron density peak is located at 0.84 Å from C5A and the deepest hole is located at 1.32 Å from C9C.

### Crystal data

**Table:**

C_18_H_19_NO_4_	*F*(000) = 1992
*M**_r_* = 313.34	*D*_x_ = 1.311 Mg m^−^^3^
Monoclinic, *P*2_1_/*c*	Melting point = 391–393 K
Hall symbol: -P 2ybc	Mo *K*α radiation, λ = 0.71073 Å
*a* = 14.8537 (3) Å	Cell parameters from 10835 reflections
*b* = 20.5009 (4) Å	θ = 1.6–27.5°
*c* = 19.5952 (3) Å	µ = 0.09 mm^−^^1^
β = 127.043 (1)°	*T* = 100 K
*V* = 4762.78 (16) Å^3^	Block, orange
*Z* = 12	0.40 × 0.20 × 0.14 mm

### Data collection

**Table:**

Bruker APEXII CCD area-detector diffractometer	10835 independent reflections
Radiation source: sealed tube	6967 reflections with *I* > 2σ(*I*)
graphite	*R*_int_ = 0.048
φ and ω scans	θ_max_ = 27.5°, θ_min_ = 1.6°
Absorption correction: multi-scan (*SADABS*; Bruker, 2005)	*h* = −16→19
*T*_min_ = 0.964, *T*_max_ = 0.987	*k* = −26→23
48383 measured reflections	*l* = −25→22

### Refinement

**Table:**

Refinement on *F*^2^	Primary atom site location: structure-invariant direct methods
Least-squares matrix: full	Secondary atom site location: difference Fourier map
*R*[*F*^2^ > 2σ(*F*^2^)] = 0.065	Hydrogen site location: inferred from neighbouring sites
*wR*(*F*^2^) = 0.124	H-atom parameters constrained
*S* = 1.02	*w* = 1/[σ^2^(*F*_o_^2^) + (0.045*P*)^2^ + 2.0308*P*] where *P* = (*F*_o_^2^ + 2*F*_c_^2^)/3
10835 reflections	(Δ/σ)_max_ = 0.001
631 parameters	Δρ_max_ = 0.29 e Å^−^^3^
0 restraints	Δρ_min_ = −0.24 e Å^−^^3^

### Special details

**Table:**

Experimental. The crystal was placed in the cold stream of an Oxford Cryosystems Cobra open-flow nitrogen cryostat (Cosier & Glazer, 1986) operating at 120.0 (1) K.
Geometry. All e.s.d.'s (except the e.s.d. in the dihedral angle between two l.s. planes) are estimated using the full covariance matrix. The cell e.s.d.'s are taken into account individually in the estimation of e.s.d.'s in distances, angles and torsion angles; correlations between e.s.d.'s in cell parameters are only used when they are defined by crystal symmetry. An approximate (isotropic) treatment of cell e.s.d.'s is used for estimating e.s.d.'s involving l.s. planes.
Refinement. Refinement of *F*^2^ against ALL reflections. The weighted *R*-factor *wR* and goodness of fit *S* are based on *F*^2^, conventional *R*-factors *R* are based on *F*, with *F* set to zero for negative *F*^2^. The threshold expression of *F*^2^ > σ(*F*^2^) is used only for calculating *R*-factors(gt) *etc*. and is not relevant to the choice of reflections for refinement. *R*-factors based on *F*^2^ are statistically about twice as large as those based on *F*, and *R*- factors based on ALL data will be even larger.

### Fractional atomic coordinates and isotropic or equivalent isotropic displacement parameters (Å^2^)

**Table:**

	*x*	*y*	*z*	*U*_iso_*/*U*_eq_	
O1A	−0.08557 (11)	0.59998 (7)	0.41896 (8)	0.0254 (3)	
O2A	0.52673 (11)	0.57793 (7)	0.68570 (9)	0.0257 (3)	
O3A	0.58623 (10)	0.54643 (6)	0.83977 (8)	0.0217 (3)	
O4A	0.43538 (11)	0.53968 (7)	0.87214 (8)	0.0261 (3)	
N1A	−0.30521 (14)	0.59162 (8)	0.32895 (10)	0.0261 (4)	
H19	−0.2434	0.5920	0.3292	0.031*	
H20	−0.3747	0.5857	0.2819	0.031*	
C1A	−0.16618 (18)	0.58769 (11)	0.56039 (13)	0.0298 (5)	
H1	−0.0930	0.5865	0.6135	0.036*	
C2A	−0.25800 (19)	0.58977 (12)	0.56076 (15)	0.0400 (6)	
H2	−0.2484	0.5914	0.6132	0.048*	
C3A	−0.36563 (19)	0.58947 (12)	0.48320 (15)	0.0382 (6)	
H3	−0.4297	0.5902	0.4830	0.046*	
C4A	−0.38029 (17)	0.58815 (10)	0.40738 (14)	0.0290 (5)	
H4	−0.4545	0.5871	0.3552	0.035*	
C5A	−0.28689 (17)	0.58837 (9)	0.40534 (13)	0.0209 (4)	
C6A	−0.17632 (16)	0.58721 (9)	0.48412 (12)	0.0200 (4)	
C7A	−0.07679 (16)	0.58959 (9)	0.48529 (12)	0.0187 (4)	
C8A	0.03760 (16)	0.58068 (9)	0.56635 (12)	0.0210 (4)	
H5	0.0462	0.5717	0.6176	0.025*	
C9A	0.12831 (16)	0.58506 (10)	0.56832 (12)	0.0221 (5)	
H6	0.1144	0.5951	0.5154	0.027*	
C10A	0.24653 (16)	0.57655 (9)	0.64106 (12)	0.0195 (4)	
C11A	0.32747 (16)	0.58125 (9)	0.62651 (13)	0.0217 (5)	
H11A	0.3048	0.5905	0.5707	0.026*	
C12A	0.44065 (16)	0.57256 (9)	0.69308 (13)	0.0197 (4)	
C13A	0.47368 (15)	0.55830 (9)	0.77447 (12)	0.0188 (4)	
C14A	0.39307 (16)	0.55402 (9)	0.78950 (12)	0.0194 (4)	
C15A	0.28019 (16)	0.56340 (9)	0.72344 (12)	0.0212 (5)	
H15A	0.2256	0.5609	0.7339	0.025*	
C16A	0.49555 (19)	0.59344 (11)	0.60286 (14)	0.0324 (5)	
H16A	0.5637	0.5985	0.6062	0.049*	
H16B	0.4526	0.6343	0.5827	0.049*	
H16C	0.4491	0.5582	0.5629	0.049*	
C17A	0.64119 (18)	0.60101 (10)	0.89646 (14)	0.0310 (5)	
H17A	0.7197	0.5897	0.9424	0.047*	
H17B	0.6021	0.6122	0.9211	0.047*	
H17C	0.6393	0.6385	0.8646	0.047*	
C18A	0.35598 (18)	0.53242 (14)	0.88995 (14)	0.0434 (7)	
H18A	0.3962	0.5245	0.9512	0.065*	
H18B	0.3061	0.4955	0.8574	0.065*	
H18C	0.3110	0.5723	0.8735	0.065*	
O1B	0.08702 (11)	0.85840 (7)	0.53926 (9)	0.0263 (3)	
O2B	0.38583 (13)	0.68996 (8)	0.39865 (10)	0.0442 (4)	
O3B	0.24409 (14)	0.59389 (8)	0.30000 (10)	0.0420 (4)	
O4B	0.06198 (13)	0.56659 (7)	0.29336 (9)	0.0312 (4)	
N1B	0.01004 (16)	0.88768 (9)	0.62644 (12)	0.0355 (5)	
H21	0.0618	0.8949	0.6190	0.043*	
H22	0.0069	0.9101	0.6625	0.043*	
C1B	−0.15505 (16)	0.75738 (10)	0.46919 (13)	0.0235 (5)	
H7	−0.1558	0.7316	0.4286	0.028*	
C2B	−0.23981 (17)	0.74915 (10)	0.47736 (14)	0.0282 (5)	
H8	−0.2990	0.7189	0.4421	0.034*	
C3B	−0.23785 (17)	0.78576 (10)	0.53797 (13)	0.0275 (5)	
H9	−0.2950	0.7796	0.5452	0.033*	
C4B	−0.15435 (17)	0.83070 (10)	0.58735 (13)	0.0257 (5)	
H10	−0.1545	0.8551	0.6285	0.031*	
C5B	−0.06833 (16)	0.84142 (10)	0.57843 (12)	0.0218 (5)	
C6B	−0.06701 (16)	0.80257 (9)	0.51860 (12)	0.0194 (4)	
C7B	0.02390 (16)	0.80988 (10)	0.50969 (12)	0.0199 (4)	
C8B	0.04421 (16)	0.75847 (10)	0.46772 (12)	0.0212 (5)	
H11	−0.0085	0.7239	0.4389	0.025*	
C9B	0.13545 (17)	0.75989 (10)	0.46976 (12)	0.0228 (5)	
H12	0.1867	0.7948	0.5008	0.027*	
C10B	0.16565 (16)	0.71376 (10)	0.42958 (12)	0.0221 (5)	
C11B	0.26417 (17)	0.72409 (11)	0.43726 (13)	0.0283 (5)	
H11B	0.3124	0.7594	0.4708	0.034*	
C12B	0.29238 (18)	0.68327 (11)	0.39632 (13)	0.0301 (5)	
C13B	0.22229 (18)	0.63080 (11)	0.34738 (13)	0.0288 (5)	
C14B	0.12561 (17)	0.61928 (10)	0.34194 (13)	0.0246 (5)	
C15B	0.09686 (17)	0.66057 (10)	0.38183 (12)	0.0228 (5)	
H15B	0.0300	0.6528	0.3768	0.027*	
C16B	0.4517 (2)	0.74717 (15)	0.43696 (18)	0.0567 (8)	
H16D	0.5096	0.7488	0.4278	0.085*	
H16E	0.4879	0.7466	0.4985	0.085*	
H16F	0.4030	0.7856	0.4111	0.085*	
C17B	0.3097 (2)	0.53913 (13)	0.33892 (17)	0.0558 (8)	
H17D	0.3303	0.5212	0.3037	0.084*	
H17E	0.2672	0.5064	0.3454	0.084*	
H17F	0.3783	0.5508	0.3953	0.084*	
C18B	−0.0306 (2)	0.54963 (11)	0.29440 (15)	0.0357 (6)	
H18D	−0.0659	0.5095	0.2615	0.054*	
H18E	−0.0861	0.5850	0.2688	0.054*	
H18F	−0.0031	0.5428	0.3536	0.054*	
O1C	0.61005 (12)	0.66222 (7)	0.14192 (10)	0.0341 (4)	
O2C	1.10540 (11)	0.85256 (7)	0.27075 (9)	0.0254 (3)	
O3C	1.10744 (11)	0.95187 (7)	0.36002 (8)	0.0249 (3)	
O4C	0.94560 (11)	0.96655 (6)	0.38012 (9)	0.0246 (3)	
N1C	0.45888 (15)	0.58972 (9)	0.13558 (12)	0.0347 (5)	
H23	0.5094	0.5926	0.1224	0.042*	
H24	0.4258	0.5552	0.1314	0.042*	
C1C	0.46971 (17)	0.75639 (10)	0.21002 (13)	0.0274 (5)	
H13	0.5096	0.7962	0.2222	0.033*	
C2C	0.38575 (18)	0.75371 (11)	0.22009 (14)	0.0326 (5)	
H14	0.3670	0.7912	0.2377	0.039*	
C3C	0.32890 (18)	0.69528 (11)	0.20407 (14)	0.0330 (6)	
H15	0.2723	0.6924	0.2124	0.040*	
C4C	0.35370 (17)	0.64185 (11)	0.17648 (14)	0.0305 (5)	
H16	0.3139	0.6023	0.1661	0.037*	
C5C	0.43660 (16)	0.64397 (10)	0.16316 (13)	0.0247 (5)	
C6C	0.49882 (16)	0.70271 (10)	0.18239 (13)	0.0231 (5)	
C7C	0.59376 (17)	0.70559 (10)	0.17751 (13)	0.0243 (5)	
C8C	0.67448 (17)	0.76082 (10)	0.21832 (13)	0.0251 (5)	
H17	0.6606	0.7955	0.2431	0.030*	
C9C	0.76553 (16)	0.76249 (10)	0.22076 (12)	0.0222 (5)	
H18	0.7752	0.7266	0.1951	0.027*	
C10C	0.85310 (16)	0.81274 (9)	0.25809 (12)	0.0201 (4)	
C11C	0.93685 (16)	0.80714 (10)	0.24673 (12)	0.0212 (5)	
H11C	0.9360	0.7712	0.2157	0.025*	
C12C	1.02123 (16)	0.85350 (10)	0.28036 (12)	0.0201 (4)	
C13C	1.02328 (16)	0.90582 (9)	0.32670 (12)	0.0193 (4)	
C14C	0.93758 (16)	0.91248 (9)	0.33641 (12)	0.0190 (4)	
C15C	0.85315 (16)	0.86616 (10)	0.30274 (12)	0.0205 (4)	
H15C	0.7956	0.8705	0.3098	0.025*	
C16C	1.10465 (18)	0.79886 (10)	0.22338 (14)	0.0288 (5)	
H16G	1.1680	0.8032	0.2205	0.043*	
H16H	1.0336	0.7989	0.1654	0.043*	
H16I	1.1118	0.7578	0.2519	0.043*	
C17C	1.1897 (2)	0.94771 (14)	0.45019 (14)	0.0450 (7)	
H17G	1.2346	0.9879	0.4715	0.068*	
H17H	1.2393	0.9104	0.4642	0.068*	
H17I	1.1520	0.9420	0.4771	0.068*	
C18C	0.86882 (17)	0.97201 (10)	0.40105 (14)	0.0262 (5)	
H18G	0.8839	1.0125	0.4329	0.039*	
H18H	0.8786	0.9347	0.4362	0.039*	
H18I	0.7913	0.9726	0.3484	0.039*	

### Atomic displacement parameters (Å^2^)

**Table:**

	*U*^11^	*U*^22^	*U*^33^	*U*^12^	*U*^13^	*U*^23^
O1A	0.0228 (7)	0.0336 (9)	0.0183 (7)	0.0006 (6)	0.0115 (7)	0.0015 (7)
O2A	0.0243 (8)	0.0322 (9)	0.0282 (8)	0.0024 (6)	0.0198 (7)	0.0040 (7)
O3A	0.0157 (7)	0.0215 (8)	0.0240 (8)	0.0003 (6)	0.0099 (6)	−0.0021 (6)
O4A	0.0186 (7)	0.0402 (9)	0.0204 (7)	0.0043 (6)	0.0122 (6)	0.0058 (7)
N1A	0.0178 (9)	0.0356 (11)	0.0172 (9)	−0.0010 (8)	0.0064 (8)	−0.0008 (8)
C1A	0.0239 (11)	0.0403 (15)	0.0217 (11)	−0.0081 (10)	0.0119 (10)	−0.0038 (11)
C2A	0.0361 (14)	0.0633 (18)	0.0298 (13)	−0.0146 (12)	0.0248 (12)	−0.0102 (12)
C3A	0.0301 (13)	0.0533 (17)	0.0401 (14)	−0.0135 (12)	0.0258 (12)	−0.0116 (13)
C4A	0.0183 (11)	0.0329 (14)	0.0296 (12)	−0.0082 (10)	0.0110 (10)	−0.0068 (11)
C5A	0.0243 (11)	0.0125 (11)	0.0240 (11)	−0.0028 (9)	0.0136 (10)	−0.0029 (9)
C6A	0.0223 (11)	0.0166 (11)	0.0204 (11)	−0.0029 (9)	0.0125 (9)	−0.0025 (9)
C7A	0.0238 (11)	0.0105 (11)	0.0208 (11)	−0.0019 (8)	0.0129 (9)	−0.0018 (9)
C8A	0.0245 (11)	0.0191 (12)	0.0179 (10)	−0.0002 (9)	0.0120 (9)	0.0015 (9)
C9A	0.0239 (11)	0.0238 (12)	0.0187 (10)	0.0018 (9)	0.0129 (9)	0.0017 (9)
C10A	0.0210 (10)	0.0151 (11)	0.0209 (11)	0.0012 (8)	0.0119 (9)	0.0006 (9)
C11A	0.0258 (11)	0.0214 (12)	0.0205 (11)	0.0021 (9)	0.0154 (10)	0.0022 (9)
C12A	0.0211 (11)	0.0153 (11)	0.0270 (11)	0.0001 (9)	0.0168 (10)	−0.0014 (9)
C13A	0.0174 (10)	0.0128 (11)	0.0227 (11)	0.0007 (8)	0.0102 (9)	−0.0005 (9)
C14A	0.0213 (10)	0.0175 (11)	0.0187 (10)	0.0011 (9)	0.0117 (9)	0.0008 (9)
C15A	0.0199 (10)	0.0206 (12)	0.0259 (11)	0.0005 (9)	0.0153 (10)	0.0012 (9)
C16A	0.0343 (13)	0.0419 (15)	0.0320 (13)	0.0026 (11)	0.0257 (11)	0.0042 (11)
C17A	0.0226 (11)	0.0274 (13)	0.0328 (13)	−0.0062 (10)	0.0112 (10)	−0.0104 (11)
C18A	0.0260 (12)	0.081 (2)	0.0289 (13)	0.0116 (13)	0.0196 (11)	0.0183 (13)
O1B	0.0258 (8)	0.0237 (9)	0.0293 (8)	−0.0047 (7)	0.0166 (7)	−0.0044 (7)
O2B	0.0310 (9)	0.0638 (12)	0.0452 (10)	−0.0059 (9)	0.0269 (8)	−0.0117 (9)
O3B	0.0540 (11)	0.0458 (11)	0.0364 (9)	0.0192 (9)	0.0327 (9)	0.0067 (8)
O4B	0.0477 (10)	0.0257 (9)	0.0322 (9)	−0.0040 (7)	0.0304 (8)	−0.0053 (7)
N1B	0.0460 (12)	0.0374 (12)	0.0363 (11)	−0.0174 (10)	0.0318 (10)	−0.0177 (10)
C1B	0.0231 (11)	0.0168 (12)	0.0256 (11)	0.0028 (9)	0.0120 (10)	0.0003 (9)
C2B	0.0225 (11)	0.0205 (12)	0.0348 (13)	−0.0010 (9)	0.0137 (10)	0.0010 (10)
C3B	0.0258 (12)	0.0275 (13)	0.0307 (12)	0.0040 (10)	0.0178 (11)	0.0087 (11)
C4B	0.0294 (12)	0.0285 (13)	0.0214 (11)	0.0027 (10)	0.0165 (10)	0.0044 (10)
C5B	0.0243 (11)	0.0195 (12)	0.0179 (10)	0.0027 (9)	0.0106 (9)	0.0051 (9)
C6B	0.0219 (10)	0.0145 (11)	0.0185 (10)	0.0032 (9)	0.0105 (9)	0.0043 (9)
C7B	0.0197 (10)	0.0177 (12)	0.0161 (10)	0.0015 (9)	0.0075 (9)	0.0039 (9)
C8B	0.0235 (11)	0.0174 (12)	0.0200 (11)	−0.0025 (9)	0.0117 (9)	−0.0001 (9)
C9B	0.0240 (11)	0.0209 (12)	0.0183 (11)	−0.0028 (9)	0.0100 (9)	−0.0001 (9)
C10B	0.0245 (11)	0.0238 (12)	0.0169 (10)	0.0033 (9)	0.0119 (9)	0.0051 (9)
C11B	0.0241 (11)	0.0338 (14)	0.0227 (11)	−0.0010 (10)	0.0118 (10)	−0.0010 (10)
C12B	0.0254 (12)	0.0411 (15)	0.0270 (12)	0.0058 (11)	0.0176 (10)	0.0050 (11)
C13B	0.0333 (12)	0.0341 (14)	0.0236 (11)	0.0106 (11)	0.0195 (11)	0.0054 (11)
C14B	0.0329 (12)	0.0226 (13)	0.0182 (11)	0.0030 (10)	0.0154 (10)	0.0035 (10)
C15B	0.0265 (11)	0.0229 (12)	0.0203 (11)	0.0028 (9)	0.0148 (10)	0.0047 (9)
C16B	0.0348 (14)	0.090 (2)	0.0536 (17)	−0.0210 (15)	0.0311 (14)	−0.0245 (17)
C17B	0.0668 (19)	0.0480 (18)	0.0478 (17)	0.0259 (15)	0.0320 (16)	0.0070 (14)
C18B	0.0551 (15)	0.0293 (14)	0.0389 (14)	−0.0165 (12)	0.0369 (13)	−0.0114 (11)
O1C	0.0397 (9)	0.0287 (9)	0.0403 (9)	−0.0107 (7)	0.0275 (8)	−0.0132 (8)
O2C	0.0274 (8)	0.0291 (9)	0.0277 (8)	−0.0049 (6)	0.0209 (7)	−0.0052 (7)
O3C	0.0274 (8)	0.0257 (8)	0.0228 (8)	−0.0103 (7)	0.0158 (7)	−0.0026 (6)
O4C	0.0302 (8)	0.0190 (8)	0.0325 (8)	−0.0048 (6)	0.0230 (7)	−0.0066 (7)
N1C	0.0271 (10)	0.0202 (11)	0.0463 (12)	−0.0064 (8)	0.0166 (10)	−0.0038 (9)
C1C	0.0246 (11)	0.0222 (13)	0.0304 (12)	−0.0056 (9)	0.0139 (10)	−0.0011 (10)
C2C	0.0299 (12)	0.0305 (14)	0.0392 (14)	−0.0020 (11)	0.0217 (11)	−0.0032 (11)
C3C	0.0235 (12)	0.0404 (15)	0.0328 (13)	−0.0018 (11)	0.0157 (11)	0.0056 (11)
C4C	0.0192 (11)	0.0266 (13)	0.0318 (13)	−0.0057 (10)	0.0080 (10)	0.0064 (11)
C5C	0.0174 (10)	0.0201 (12)	0.0211 (11)	0.0005 (9)	0.0035 (9)	0.0055 (10)
C6C	0.0208 (11)	0.0197 (12)	0.0216 (11)	−0.0010 (9)	0.0089 (9)	0.0016 (9)
C7C	0.0254 (11)	0.0213 (12)	0.0213 (11)	0.0005 (9)	0.0114 (10)	0.0019 (10)
C8C	0.0272 (11)	0.0209 (12)	0.0262 (12)	−0.0044 (9)	0.0156 (10)	−0.0046 (10)
C9C	0.0267 (11)	0.0213 (12)	0.0189 (11)	−0.0029 (9)	0.0139 (10)	−0.0031 (9)
C10C	0.0223 (10)	0.0195 (12)	0.0153 (10)	−0.0015 (9)	0.0096 (9)	0.0016 (9)
C11C	0.0260 (11)	0.0209 (12)	0.0168 (10)	−0.0006 (9)	0.0130 (9)	−0.0025 (9)
C12C	0.0216 (10)	0.0241 (12)	0.0160 (10)	−0.0003 (9)	0.0121 (9)	0.0031 (9)
C13C	0.0223 (11)	0.0182 (11)	0.0163 (10)	−0.0030 (9)	0.0111 (9)	0.0027 (9)
C14C	0.0243 (11)	0.0153 (11)	0.0163 (10)	−0.0002 (9)	0.0117 (9)	0.0016 (9)
C15C	0.0215 (10)	0.0218 (12)	0.0191 (10)	0.0007 (9)	0.0128 (9)	0.0020 (9)
C16C	0.0344 (12)	0.0272 (13)	0.0325 (12)	0.0009 (10)	0.0242 (11)	−0.0022 (10)
C17C	0.0392 (14)	0.0702 (19)	0.0240 (13)	−0.0283 (13)	0.0182 (12)	−0.0130 (13)
C18C	0.0311 (12)	0.0221 (12)	0.0326 (12)	−0.0010 (10)	0.0230 (11)	−0.0035 (10)

### Geometric parameters (Å, °)

**Table:**

O1A—C7A	1.244 (2)	C8B—C9B	1.332 (3)
O2A—C12A	1.375 (2)	C8B—H11	0.9500
O2A—C16A	1.430 (2)	C9B—C10B	1.463 (3)
O3A—C13A	1.384 (2)	C9B—H12	0.9500
O3A—C17A	1.435 (2)	C10B—C11B	1.394 (3)
O4A—C14A	1.370 (2)	C10B—C15B	1.399 (3)
O4A—C18A	1.425 (2)	C11B—C12B	1.387 (3)
N1A—C5A	1.352 (2)	C11B—H11B	0.9500
N1A—H19	0.9142	C12B—C13B	1.399 (3)
N1A—H20	0.8852	C13B—C14B	1.395 (3)
C1A—C2A	1.369 (3)	C14B—C15B	1.383 (3)
C1A—C6A	1.409 (3)	C15B—H15B	0.9500
C1A—H1	0.9500	C16B—H16D	0.9800
C2A—C3A	1.392 (3)	C16B—H16E	0.9800
C2A—H2	0.9500	C16B—H16F	0.9800
C3A—C4A	1.366 (3)	C17B—H17D	0.9800
C3A—H3	0.9500	C17B—H17E	0.9800
C4A—C5A	1.412 (3)	C17B—H17F	0.9800
C4A—H4	0.9500	C18B—H18D	0.9800
C5A—C6A	1.424 (3)	C18B—H18E	0.9800
C6A—C7A	1.466 (3)	C18B—H18F	0.9800
C7A—C8A	1.480 (3)	O1C—C7C	1.241 (2)
C8A—C9A	1.328 (3)	O2C—C12C	1.372 (2)
C8A—H5	0.9500	O2C—C16C	1.436 (2)
C9A—C10A	1.460 (3)	O3C—C13C	1.377 (2)
C9A—H6	0.9500	O3C—C17C	1.422 (3)
C10A—C11A	1.396 (3)	O4C—C14C	1.361 (2)
C10A—C15A	1.399 (3)	O4C—C18C	1.430 (2)
C11A—C12A	1.385 (3)	N1C—C5C	1.362 (3)
C11A—H11A	0.9500	N1C—H23	0.9303
C12A—C13A	1.388 (3)	N1C—H24	0.8372
C13A—C14A	1.397 (3)	C1C—C2C	1.376 (3)
C14A—C15A	1.382 (3)	C1C—C6C	1.405 (3)
C15A—H15A	0.9500	C1C—H13	0.9500
C16A—H16A	0.9800	C2C—C3C	1.388 (3)
C16A—H16B	0.9800	C2C—H14	0.9500
C16A—H16C	0.9800	C3C—C4C	1.367 (3)
C17A—H17A	0.9800	C3C—H15	0.9500
C17A—H17B	0.9800	C4C—C5C	1.405 (3)
C17A—H17C	0.9800	C4C—H16	0.9500
C18A—H18A	0.9800	C5C—C6C	1.424 (3)
C18A—H18B	0.9800	C6C—C7C	1.471 (3)
C18A—H18C	0.9800	C7C—C8C	1.484 (3)
O1B—C7B	1.245 (2)	C8C—C9C	1.325 (3)
O2B—C12B	1.368 (2)	C8C—H17	0.9500
O2B—C16B	1.418 (3)	C9C—C10C	1.464 (3)
O3B—C17B	1.377 (3)	C9C—H18	0.9500
O3B—C13B	1.379 (2)	C10C—C11C	1.394 (3)
O4B—C14B	1.372 (2)	C10C—C15C	1.401 (3)
O4B—C18B	1.430 (2)	C11C—C12C	1.383 (3)
N1B—C5B	1.349 (3)	C11C—H11C	0.9500
N1B—H21	0.8767	C12C—C13C	1.394 (3)
N1B—H22	0.8666	C13C—C14C	1.403 (3)
C1B—C2B	1.372 (3)	C14C—C15C	1.383 (3)
C1B—C6B	1.407 (3)	C15C—H15C	0.9500
C1B—H7	0.9500	C16C—H16G	0.9800
C2B—C3B	1.390 (3)	C16C—H16H	0.9800
C2B—H8	0.9500	C16C—H16I	0.9800
C3B—C4B	1.370 (3)	C17C—H17G	0.9800
C3B—H9	0.9500	C17C—H17H	0.9800
C4B—C5B	1.407 (3)	C17C—H17I	0.9800
C4B—H10	0.9500	C18C—H18G	0.9800
C5B—C6B	1.427 (3)	C18C—H18H	0.9800
C6B—C7B	1.470 (3)	C18C—H18I	0.9800
C7B—C8B	1.476 (3)		
			
C12A—O2A—C16A	116.84 (15)	C15B—C10B—C9B	121.77 (18)
C13A—O3A—C17A	112.68 (14)	C12B—C11B—C10B	120.6 (2)
C14A—O4A—C18A	116.99 (15)	C12B—C11B—H11B	119.7
C5A—N1A—H19	117.5	C10B—C11B—H11B	119.7
C5A—N1A—H20	118.5	O2B—C12B—C11B	125.0 (2)
H19—N1A—H20	123.0	O2B—C12B—C13B	114.90 (18)
C2A—C1A—C6A	122.4 (2)	C11B—C12B—C13B	120.06 (19)
C2A—C1A—H1	118.8	O3B—C13B—C14B	120.3 (2)
C6A—C1A—H1	118.8	O3B—C13B—C12B	120.25 (19)
C1A—C2A—C3A	119.1 (2)	C14B—C13B—C12B	119.29 (18)
C1A—C2A—H2	120.4	O4B—C14B—C15B	123.78 (18)
C3A—C2A—H2	120.4	O4B—C14B—C13B	115.69 (18)
C4A—C3A—C2A	120.9 (2)	C15B—C14B—C13B	120.5 (2)
C4A—C3A—H3	119.6	C14B—C15B—C10B	120.36 (19)
C2A—C3A—H3	119.6	C14B—C15B—H15B	119.8
C3A—C4A—C5A	121.0 (2)	C10B—C15B—H15B	119.8
C3A—C4A—H4	119.5	O2B—C16B—H16D	109.5
C5A—C4A—H4	119.5	O2B—C16B—H16E	109.5
N1A—C5A—C4A	119.06 (18)	H16D—C16B—H16E	109.5
N1A—C5A—C6A	122.18 (17)	O2B—C16B—H16F	109.5
C4A—C5A—C6A	118.73 (18)	H16D—C16B—H16F	109.5
C1A—C6A—C5A	117.81 (17)	H16E—C16B—H16F	109.5
C1A—C6A—C7A	121.40 (17)	O3B—C17B—H17D	109.5
C5A—C6A—C7A	120.68 (17)	O3B—C17B—H17E	109.5
O1A—C7A—C6A	121.31 (17)	H17D—C17B—H17E	109.5
O1A—C7A—C8A	118.18 (17)	O3B—C17B—H17F	109.5
C6A—C7A—C8A	120.50 (17)	H17D—C17B—H17F	109.5
C9A—C8A—C7A	120.76 (18)	H17E—C17B—H17F	109.5
C9A—C8A—H5	119.6	O4B—C18B—H18D	109.5
C7A—C8A—H5	119.6	O4B—C18B—H18E	109.5
C8A—C9A—C10A	128.55 (18)	H18D—C18B—H18E	109.5
C8A—C9A—H6	115.7	O4B—C18B—H18F	109.5
C10A—C9A—H6	115.7	H18D—C18B—H18F	109.5
C11A—C10A—C15A	119.74 (17)	H18E—C18B—H18F	109.5
C11A—C10A—C9A	118.01 (17)	C12C—O2C—C16C	116.54 (15)
C15A—C10A—C9A	122.24 (17)	C13C—O3C—C17C	113.79 (15)
C12A—C11A—C10A	120.32 (18)	C14C—O4C—C18C	117.49 (15)
C12A—C11A—H11A	119.8	C5C—N1C—H23	119.1
C10A—C11A—H11A	119.8	C5C—N1C—H24	117.5
O2A—C12A—C11A	124.66 (17)	H23—N1C—H24	123.4
O2A—C12A—C13A	115.49 (16)	C2C—C1C—C6C	122.7 (2)
C11A—C12A—C13A	119.85 (17)	C2C—C1C—H13	118.7
O3A—C13A—C12A	119.56 (16)	C6C—C1C—H13	118.7
O3A—C13A—C14A	120.36 (17)	C1C—C2C—C3C	118.8 (2)
C12A—C13A—C14A	120.05 (17)	C1C—C2C—H14	120.6
O4A—C14A—C15A	124.81 (17)	C3C—C2C—H14	120.6
O4A—C14A—C13A	114.92 (16)	C4C—C3C—C2C	120.6 (2)
C15A—C14A—C13A	120.27 (17)	C4C—C3C—H15	119.7
C14A—C15A—C10A	119.76 (17)	C2C—C3C—H15	119.7
C14A—C15A—H15A	120.1	C3C—C4C—C5C	121.6 (2)
C10A—C15A—H15A	120.1	C3C—C4C—H16	119.2
O2A—C16A—H16A	109.5	C5C—C4C—H16	119.2
O2A—C16A—H16B	109.5	N1C—C5C—C4C	119.88 (19)
H16A—C16A—H16B	109.5	N1C—C5C—C6C	121.45 (18)
O2A—C16A—H16C	109.5	C4C—C5C—C6C	118.62 (19)
H16A—C16A—H16C	109.5	C1C—C6C—C5C	117.59 (18)
H16B—C16A—H16C	109.5	C1C—C6C—C7C	121.55 (18)
O3A—C17A—H17A	109.5	C5C—C6C—C7C	120.78 (18)
O3A—C17A—H17B	109.5	O1C—C7C—C6C	121.81 (18)
H17A—C17A—H17B	109.5	O1C—C7C—C8C	118.80 (18)
O3A—C17A—H17C	109.5	C6C—C7C—C8C	119.37 (18)
H17A—C17A—H17C	109.5	C9C—C8C—C7C	120.99 (19)
H17B—C17A—H17C	109.5	C9C—C8C—H17	119.5
O4A—C18A—H18A	109.5	C7C—C8C—H17	119.5
O4A—C18A—H18B	109.5	C8C—C9C—C10C	128.09 (19)
H18A—C18A—H18B	109.5	C8C—C9C—H18	116.0
O4A—C18A—H18C	109.5	C10C—C9C—H18	116.0
H18A—C18A—H18C	109.5	C11C—C10C—C15C	119.94 (17)
H18B—C18A—H18C	109.5	C11C—C10C—C9C	118.16 (17)
C12B—O2B—C16B	117.57 (18)	C15C—C10C—C9C	121.89 (17)
C17B—O3B—C13B	116.71 (18)	C12C—C11C—C10C	120.45 (18)
C14B—O4B—C18B	116.60 (15)	C12C—C11C—H11C	119.8
C5B—N1B—H21	118.5	C10C—C11C—H11C	119.8
C5B—N1B—H22	119.6	O2C—C12C—C11C	124.33 (17)
H21—N1B—H22	121.9	O2C—C12C—C13C	115.86 (16)
C2B—C1B—C6B	122.59 (19)	C11C—C12C—C13C	119.81 (17)
C2B—C1B—H7	118.7	O3C—C13C—C12C	119.59 (16)
C6B—C1B—H7	118.7	O3C—C13C—C14C	120.51 (17)
C1B—C2B—C3B	119.0 (2)	C12C—C13C—C14C	119.86 (17)
C1B—C2B—H8	120.5	O4C—C14C—C15C	124.73 (17)
C3B—C2B—H8	120.5	O4C—C14C—C13C	114.97 (16)
C4B—C3B—C2B	120.69 (19)	C15C—C14C—C13C	120.31 (17)
C4B—C3B—H9	119.7	C14C—C15C—C10C	119.58 (17)
C2B—C3B—H9	119.7	C14C—C15C—H15C	120.2
C3B—C4B—C5B	121.38 (19)	C10C—C15C—H15C	120.2
C3B—C4B—H10	119.3	O2C—C16C—H16G	109.5
C5B—C4B—H10	119.3	O2C—C16C—H16H	109.5
N1B—C5B—C4B	119.33 (18)	H16G—C16C—H16H	109.5
N1B—C5B—C6B	122.14 (18)	O2C—C16C—H16I	109.5
C4B—C5B—C6B	118.53 (18)	H16G—C16C—H16I	109.5
C1B—C6B—C5B	117.74 (17)	H16H—C16C—H16I	109.5
C1B—C6B—C7B	121.67 (18)	O3C—C17C—H17G	109.5
C5B—C6B—C7B	120.59 (17)	O3C—C17C—H17H	109.5
O1B—C7B—C6B	120.96 (17)	H17G—C17C—H17H	109.5
O1B—C7B—C8B	118.73 (17)	O3C—C17C—H17I	109.5
C6B—C7B—C8B	120.28 (17)	H17G—C17C—H17I	109.5
C9B—C8B—C7B	120.91 (19)	H17H—C17C—H17I	109.5
C9B—C8B—H11	119.5	O4C—C18C—H18G	109.5
C7B—C8B—H11	119.5	O4C—C18C—H18H	109.5
C8B—C9B—C10B	127.30 (19)	H18G—C18C—H18H	109.5
C8B—C9B—H12	116.3	O4C—C18C—H18I	109.5
C10B—C9B—H12	116.3	H18G—C18C—H18I	109.5
C11B—C10B—C15B	119.14 (18)	H18H—C18C—H18I	109.5
C11B—C10B—C9B	119.07 (19)		
			
C6A—C1A—C2A—C3A	1.9 (4)	C9B—C10B—C11B—C12B	−176.66 (19)
C1A—C2A—C3A—C4A	−1.0 (4)	C16B—O2B—C12B—C11B	−8.1 (3)
C2A—C3A—C4A—C5A	−1.3 (4)	C16B—O2B—C12B—C13B	171.3 (2)
C3A—C4A—C5A—N1A	−175.4 (2)	C10B—C11B—C12B—O2B	178.81 (19)
C3A—C4A—C5A—C6A	2.6 (3)	C10B—C11B—C12B—C13B	−0.6 (3)
C2A—C1A—C6A—C5A	−0.6 (3)	C17B—O3B—C13B—C14B	−92.2 (3)
C2A—C1A—C6A—C7A	175.7 (2)	C17B—O3B—C13B—C12B	92.6 (3)
N1A—C5A—C6A—C1A	176.31 (19)	O2B—C12B—C13B—O3B	−5.7 (3)
C4A—C5A—C6A—C1A	−1.7 (3)	C11B—C12B—C13B—O3B	173.78 (19)
N1A—C5A—C6A—C7A	0.1 (3)	O2B—C12B—C13B—C14B	179.11 (18)
C4A—C5A—C6A—C7A	−177.94 (18)	C11B—C12B—C13B—C14B	−1.4 (3)
C1A—C6A—C7A—O1A	−167.99 (19)	C18B—O4B—C14B—C15B	−8.4 (3)
C5A—C6A—C7A—O1A	8.1 (3)	C18B—O4B—C14B—C13B	173.43 (18)
C1A—C6A—C7A—C8A	11.0 (3)	O3B—C13B—C14B—O4B	5.3 (3)
C5A—C6A—C7A—C8A	−172.87 (18)	C12B—C13B—C14B—O4B	−179.45 (18)
O1A—C7A—C8A—C9A	1.4 (3)	O3B—C13B—C14B—C15B	−172.86 (18)
C6A—C7A—C8A—C9A	−177.63 (18)	C12B—C13B—C14B—C15B	2.4 (3)
C7A—C8A—C9A—C10A	−178.66 (19)	O4B—C14B—C15B—C10B	−179.30 (18)
C8A—C9A—C10A—C11A	177.7 (2)	C13B—C14B—C15B—C10B	−1.3 (3)
C8A—C9A—C10A—C15A	−1.7 (3)	C11B—C10B—C15B—C14B	−0.8 (3)
C15A—C10A—C11A—C12A	0.4 (3)	C9B—C10B—C15B—C14B	177.52 (18)
C9A—C10A—C11A—C12A	−178.99 (18)	C6C—C1C—C2C—C3C	1.4 (3)
C16A—O2A—C12A—C11A	−0.2 (3)	C1C—C2C—C3C—C4C	−1.8 (3)
C16A—O2A—C12A—C13A	−179.21 (17)	C2C—C3C—C4C—C5C	−0.2 (3)
C10A—C11A—C12A—O2A	−178.18 (18)	C3C—C4C—C5C—N1C	−180.0 (2)
C10A—C11A—C12A—C13A	0.8 (3)	C3C—C4C—C5C—C6C	2.6 (3)
C17A—O3A—C13A—C12A	102.8 (2)	C2C—C1C—C6C—C5C	1.0 (3)
C17A—O3A—C13A—C14A	−79.2 (2)	C2C—C1C—C6C—C7C	−176.0 (2)
O2A—C12A—C13A—O3A	−4.2 (3)	N1C—C5C—C6C—C1C	179.67 (18)
C11A—C12A—C13A—O3A	176.75 (17)	C4C—C5C—C6C—C1C	−2.9 (3)
O2A—C12A—C13A—C14A	177.80 (17)	N1C—C5C—C6C—C7C	−3.4 (3)
C11A—C12A—C13A—C14A	−1.3 (3)	C4C—C5C—C6C—C7C	174.08 (18)
C18A—O4A—C14A—C15A	1.7 (3)	C1C—C6C—C7C—O1C	−169.5 (2)
C18A—O4A—C14A—C13A	−177.75 (19)	C5C—C6C—C7C—O1C	13.7 (3)
O3A—C13A—C14A—O4A	2.0 (3)	C1C—C6C—C7C—C8C	12.3 (3)
C12A—C13A—C14A—O4A	179.98 (17)	C5C—C6C—C7C—C8C	−164.54 (18)
O3A—C13A—C14A—C15A	−177.48 (17)	O1C—C7C—C8C—C9C	−3.4 (3)
C12A—C13A—C14A—C15A	0.5 (3)	C6C—C7C—C8C—C9C	174.90 (19)
O4A—C14A—C15A—C10A	−178.72 (18)	C7C—C8C—C9C—C10C	180.00 (18)
C13A—C14A—C15A—C10A	0.7 (3)	C8C—C9C—C10C—C11C	−174.5 (2)
C11A—C10A—C15A—C14A	−1.1 (3)	C8C—C9C—C10C—C15C	4.2 (3)
C9A—C10A—C15A—C14A	178.22 (18)	C15C—C10C—C11C—C12C	1.0 (3)
C6B—C1B—C2B—C3B	−1.4 (3)	C9C—C10C—C11C—C12C	179.69 (18)
C1B—C2B—C3B—C4B	1.7 (3)	C16C—O2C—C12C—C11C	−0.9 (3)
C2B—C3B—C4B—C5B	0.1 (3)	C16C—O2C—C12C—C13C	179.83 (17)
C3B—C4B—C5B—N1B	177.82 (19)	C10C—C11C—C12C—O2C	−178.58 (17)
C3B—C4B—C5B—C6B	−2.3 (3)	C10C—C11C—C12C—C13C	0.7 (3)
C2B—C1B—C6B—C5B	−0.8 (3)	C17C—O3C—C13C—C12C	−106.7 (2)
C2B—C1B—C6B—C7B	179.06 (19)	C17C—O3C—C13C—C14C	75.6 (2)
N1B—C5B—C6B—C1B	−177.55 (19)	O2C—C12C—C13C—O3C	−0.7 (3)
C4B—C5B—C6B—C1B	2.6 (3)	C11C—C12C—C13C—O3C	−179.97 (17)
N1B—C5B—C6B—C7B	2.6 (3)	O2C—C12C—C13C—C14C	177.04 (16)
C4B—C5B—C6B—C7B	−177.27 (17)	C11C—C12C—C13C—C14C	−2.3 (3)
C1B—C6B—C7B—O1B	164.52 (18)	C18C—O4C—C14C—C15C	7.4 (3)
C5B—C6B—C7B—O1B	−15.6 (3)	C18C—O4C—C14C—C13C	−172.62 (16)
C1B—C6B—C7B—C8B	−17.5 (3)	O3C—C13C—C14C—O4C	0.0 (3)
C5B—C6B—C7B—C8B	162.37 (17)	C12C—C13C—C14C—O4C	−177.71 (16)
O1B—C7B—C8B—C9B	7.6 (3)	O3C—C13C—C14C—C15C	179.92 (17)
C6B—C7B—C8B—C9B	−170.46 (18)	C12C—C13C—C14C—C15C	2.2 (3)
C7B—C8B—C9B—C10B	−178.27 (18)	O4C—C14C—C15C—C10C	179.35 (17)
C8B—C9B—C10B—C11B	178.6 (2)	C13C—C14C—C15C—C10C	−0.6 (3)
C8B—C9B—C10B—C15B	0.3 (3)	C11C—C10C—C15C—C14C	−1.0 (3)
C15B—C10B—C11B—C12B	1.7 (3)	C9C—C10C—C15C—C14C	−179.66 (18)

### Hydrogen-bond geometry (Å, °)

**Table:**

*Cg*1, *Cg*2 and *Cg*4 are the centroids of the C1A–C6A, C10A–C15A and C10–C15B rings, respectively.

**Table:**

*D*—H···*A*	*D*—H	H···*A*	*D*···*A*	*D*—H···*A*
N1A—H19···O1A	0.92	1.91	2.618 (3)	133
N1A—H20···N1C^i^	0.89	2.41	3.262 (3)	162
N1B—H21···O1B	0.88	1.96	2.634 (3)	132
N1B—H22···O4B^ii^	0.87	2.22	3.022 (3)	153
N1C—H23···O1C	0.93	1.93	2.633 (3)	130
N1C—H24···O3A^iii^	0.84	2.19	2.977 (2)	156
C15B—H15B···O1A	0.95	2.55	3.434 (3)	154
C18B—H18D···O3C^iv^	0.98	2.38	3.212 (3)	142
C18B—H18F···O1A	0.98	2.53	3.177 (3)	123
C2B—H8···Cg1	0.95	2.75	3.342 (2)	121
C2C—H14···Cg2^v^	0.95	2.94	3.674 (2)	135
C16C—H16I···Cg4^vi^	0.98	2.80	3.724 (2)	157

Symmetry codes:  (i) *x*−1, *y*, *z*; (ii) *x*, −*y*+3/2, *z*+1/2; (iii) −*x*+1, −*y*+1, −*z*+1; (iv) −*x*+1, *y*−1/2, −*z*+1/2; (v) *x*, −*y*+1/2, *z*−3/2; (vi) *x*+1, *y*, *z*.
